# Knowledge, Perception, and Practices of Hand Hygiene Among Medical and Paramedical Students at a Tertiary Care Institute in Uttarakhand

**DOI:** 10.7759/cureus.74812

**Published:** 2024-11-30

**Authors:** Himanshu Narula, Gaurav Chikara, Sulekha Nautiyal, Malvika Singh, Iva Chandola

**Affiliations:** 1 Department of Microbiology, Shri Guru Ram Rai Institute of Medical and Health Sciences, Dehradun, IND; 2 Department of Pharmacology, All India Institute of Medical Sciences, Rishikesh, Rishikesh, IND

**Keywords:** hand hygiene, hospital-acquired infections, knowledge, medical students, paramedical students

## Abstract

Background: Healthcare-associated infections or nosocomial infections are considered to be one of the leading causes of increased morbidity and mortality in patients. Hand hygiene is one of the simplest and most effective infection control measures to prevent nosocomial infections. Medical and paramedical students are the foundation of any healthcare system. So, induction and further training during the study period are required to build a robust healthcare system with responsible clinicians.

Aim: To access the knowledge, attitude, and practices of hand hygiene among medical and paramedical students at a tertiary care hospital in Uttarakhand.

Methods: A cross-sectional study was done among medical and paramedical students in a tertiary care center at Dehradun, Uttarakhand. The total number of study participants was 216, surveyed through a standard WHO-hand hygiene questionnaire. The questionnaire was circulated among students through Google Forms consisting of a total of 26 questions on demographics, knowledge, perception, and practices of hand hygiene. Statistical analyses were performed using Stata software, version 11 (Stata Corp., College Station, TX, USA).

Results: The overall knowledge score for hand hygiene in medical students was 16.73 whereas it was 15.17 among paramedical students. According to categories of knowledge it was found to be moderate in 48% (82 out of 147) of the medical students and 63.31% (43 out of 69) of the paramedical students. Both the groups perceived that hand hygiene is an important measure to prevent hospital-acquired infections whereas the practices were not so good in both the groups.

Conclusion: The present study reveals that there were knowledge gaps between medical and paramedical students regarding knowledge of hand hygiene. Overall scores were better among medical students as compared to paramedical students. Moderate knowledge scores in both groups emphasize a regular need for induction training of the students in the institute.

## Introduction

Healthcare-associated infections are a major threat to patient safety and increased mortality and morbidity. The true burden of HAI is highly unknown because of the lack of prevalence studies in developing countries. The hands of healthcare workers act as a major source of transmission of infections from one patient to another. According to guidelines of the Infectious Diseases Society of America (IDSA), on any given day, about one in 31 hospital patients has at least one healthcare-associated infection [1]. A point prevalence study done to estimate the prevalence of hospital-acquired infections in a tertiary care hospital in India reported an overall prevalence of 7% [2]. Another study done in northern India reported the infection prevalence being 36.2 infections per 1000 patient days [3].

Hand hygiene is an important and effective measure to reduce the burden of hospital-acquired infections in healthcare settings. It was in the 19th century, when Hungarian physician Ignaz Semmelweis, first advanced the idea of “hand hygiene” in medical settings. Implementation of good hand hygiene practices can have a great impact on reducing the burden of healthcare-associated infections in hospitals. Good hand hygiene practices depend upon how infection prevention and control (IPC) are implemented in hospitals and further depend upon several other factors like how frequently the IPC education and training are done, how much the ratio of doctor-to-patient or the ratio of nurse-patient is, and whether the audits and feedbacks related to IPC are done on regular basis or not. Hospital infection control committee is now an integral part of almost every hospital but the studies on the prevalence of hospital-acquired infections are still very less. The present study is an attempt to see the current knowledge, perception, and practices of medical and paramedical students regarding hand hygiene in the tertiary care hospital at Uttarakhand to further identify the areas of improvement.

## Materials and methods

Study design

A descriptive cross-sectional study was conducted at Shri Guru Ram Rai Institute of Medical and Health Sciences, Dehradun to determine knowledge, attitude, and hand hygiene practices among medical and paramedical students including nursing undergraduates.

Study duration

The cross-sectional study was done over six months, from July 2023 to December 2023.

Study material

The WHO standardized hand hygiene questionnaire was given to the study participants as a Google Forms questionnaire (see Appendices).

Inclusion criteria

All the medical graduates of the first academic year and paramedical students including nursing undergraduates of our university, who were willing to participate in the study were included.

Exclusion criteria

Those students who were not willing to participate in the study, residents, and faculty were excluded from the study.

Sample size

The sample size for the study was calculated to be 200. The questionnaire was given to a total of 216 participants.

Study unit

The study participants included in the study were mainly medical and paramedical students. Informed consent was taken before giving them the questionnaire in Google Forms.

Ethical approval

Ethical approval (Ref. No. SGRR/IEC/13/23) was provided by the Institutional Ethical Committee, Shri Guru Ram Rai Institute of Medical and Health Sciences, Dehradun, Uttarakhand.

Study tools

Study participants who were willing to enroll were included in the study. Simple random sampling was done to choose the study participants. The participants were provided with a WHO hand hygiene-standard questionnaire, which they had to fill in a Google Forms questionnaire and submit the responses instantaneously. Demographic data was assessed using six questions. Knowledge was assessed using nine questions which included multiple choice and “yes” or “no” questions. Perception towards hand hygiene was measured using eight questions. Practice was assessed in a similar way using three questions. A scoring system was used where one point was given for each correct response to knowledge, and zero was given for incorrect knowledge. A score of more than 75% was considered good, 50-74% moderate, and less than 50% poor.

Data analysis

The questionnaire was circulated among students through Google Forms and the data was entered using Microsoft Excel (Microsoft Corp., Redmond, WA, USA) sheets. The data analysis approach was as follows: Categorical data was presented in the form of frequency and percentage. The normality of the data set was checked using Kolmogorov-Smirnov (KS) test. For normally distributed variables, the data was expressed as mean ± standard deviation (SD), and categorical variables were compared using a z-test for proportion. Continuous variables were analyzed using the Student's t-test for unpaired data. All statistical tests were two-tailed, and a p-value < 0.05 was considered statistically significant. Statistical analyses were performed using Stata software, version 11 (Stata Corp., College Station, TX, USA).

## Results

Demographic variables

A total of 216 students participated in the study out of which medical students were 147 (68.1%) and paramedical students were 69 (31.9%). There were a total of 136 female students as compared to 80 male students. The mean age group of participants in the study was 21-23 years. The total number of medical students who participated in the study was 147 and the number of paramedical students was 69. The majority of them, that is, 173 (80%) had received formal training in hand hygiene and the majority, that is, 211 (97.6%) of them were aware of the hand hygiene practices. The source of information was training in the institute among 154 (71.3%) students. The details are given in Table 1.

**Table 1:** Demographic variables among study participants.

Variables	Frequency	Percentage
Gender		
Male	80	37.0%
Female	136	63.0%
Age		
18-20	80	37.0%
21-23	109	50.5%
>23	27	12.5%
Students category		
Medical students	147	68.1%
Paramedical students	69	31.9%
Have you received any formal training previously?		
No	43	19.9%
Yes	173	80.1%
Have you ever heard of hand hygiene practices?		
No	5	2.4%
Yes	211	97.6%
If yes, what was the source of information?		
Media	33	15.3%
Other	29	13.4%
Training in institute	154	71.3%

Knowledge

The overall knowledge score for hand hygiene in medical students was 16.73 whereas it was 15.17 among paramedical students. According to categories of knowledge, it was found to be moderate in 48% (82 out of 147) of the medical students and 63.31% (43 out of 69) of the paramedical students (Table 2).

**Table 2:** Knowledge score among medical and paramedical students.

Knowledge level	Medical students (147)	Paramedical students (69)	Total
Good	41 (24.26)	11 (15.94)	52 (24.07)
Moderate	82 (55.78)	43 (62.31)	125 (57.87)
Poor	24 (14.20)	15 (21.73)	39 (18.05)

Knowledge questionnaire

Medical students as compared to paramedical students were aware of the fact that the main route of cross-transmission of potentially harmful germs between patients in a healthcare facility were the hands of healthcare workers and the difference between knowledge of both the groups was statistically significant. When asked about the most frequent source of germs responsible for healthcare-associated infections both groups were not sure that germs already present on healthcare worker's hands or patients were the most common source of infection. The medical students were more aware as compared to paramedical students about the five moments of hand hygiene and most of them agreed more on the moment number 1 and 4, that is, before and after touching the patient. Both groups agreed that wearing jewelry and damaged skin increases the risk of getting an infection. The percentages of correct responses to the individual questions on hand hygiene knowledge of the two groups of students are shown in Table 3.

**Table 3:** The percentages of correct responses to the individual questions on hand hygiene knowledge of the two groups of students. * represents the p-value which is significant.

S. No.	Questions	Medical students N=147	Paramedical students N=69	P value
1.	Do you routinely use an alcohol-based hand rub for hand hygiene?	124 (84.4%)	61 (88.4%)	0.428
2.	Which of the following is the main route of cross-transmission of potentially harmful germs between patients in a healthcare facility? Correct answer (Healthcare workers’ hands when not clean)	88 (59.9%)	26 (37.7%)	0.002*
3.	What is the most frequent source of germs responsible for healthcare-associated infections? Correct answer (Germs already present on or within the patient)	34 (23.1%)	22 (31.9%)	0.171
4	Hand hygiene actions prevent the transmission of germs to the patient.			
	(Before touching a patient - Answer - Yes)	140 (95.2%)	57 (82.6%)	0.002*
	(Immediately after a risk of body fluid exposure - Answer - No)	33 (22.5%)	22 (31.9%)	0.137
	(After exposure to the immediate surroundings of a patient - Answer - No)	34 (24.1%)	22 (31.8%)	0.171
	(Immediately before a clean/aseptic procedure - Answer - Yes)	122 (83.0%)	55 (79.7%)	0.558
5.	Which of the following hand hygiene actions prevents transmission of germs to the healthcare worker?			
	(After touching a patient - Answer - Yes)	137 (93.2%)	55 (79.7%)	0.003*
	(Immediately after a risk of body fluid exposure - Answer - No)	129 (87.8%)	48 (69.6%)	0.001*
	(Immediately before a clean/aseptic procedure - Answer - No)	33 (22.4%)	19 (27.5%)	0.414
	(After exposure to the immediate surroundings of a patient - Answer - Yes)	132 (89.8%)	46 (66.7%)	0.000*
6.	Which of the following statements on alcohol-based handrub and handwashing with soap and water are true?			
	(Hand rubbing is more rapid for hand cleansing than hand washing - Answer - True)	131 (89.1%)	47 (68.1%)	0.002*
	(Hand rubbing causes skin dryness more than handwashing - Answer - False)	38 (25.9%)	37 (53.6%)	0.001*
	(Hand rubbing is more effective against germs than handwashing - Answer - True)	42 (28.6%)	28 (40.6%)	0.787
	(Handwashing and hand rubbing both are recommended methods of hand hygiene - Answer- True)	77 (52.4%)	41 (59.4%)	0.006*
7.	What is the minimal time needed for alcohol-based handrub to kill most germs on your hands? Answer - 20 sec	116 (78.9%)	35 (50.7%)	0.065
8.	Which type of hand hygiene method is required in the following situations?	118 (80.3%)	32 (46.4%)	0.004*
	(Before palpation of the abdomen - Answer - Rubbing)	99 (67.3%)	37 (53.6%)	0.687
	(Before giving an injection - Answer - Rubbing)	105 (71.4%)	44 (63.8%)	0.586
	(After emptying a bedpan - Answer- Washing)	138 (93.9%)	61 (88.4%)	0.553
	(After removing examination gloves - Answer - Rubbing/Washing)	80 (54.4%)	46 (66.7%)	0.990
	(After making a patient's bed - Answer - Rubbing)	125 (85.0%)	51 (73.9%)	0.041*
	(After visible exposure to blood - Answer - Washing)	106 (72.1%)	39 (56.5%)	0.023*
9.	Which of the following should be avoided, as associated with an increased likelihood of colonization of hands with harmful germs?			
	(Wearing jewelry) Answer - Yes	135 (91.8%)	50 (72.5%)	0.002
	(Damaged skin) Answer - Yes	131 (89.1%)	53 (76.80%)	0.017*
	(Artificial fingernails) Answer - Yes	44 (29.9%)	29 (42.0%)	0.797
	(Regular use of a hand cream) Answer - No	70 (52.4%)	38 (55.1%)	0.307

Perception questionnaire

There was a slight difference between the perception of medical and paramedical students regarding the impact of hand hygiene practices on a patient’s clinical outcome. The mean scores were 6.6 and 5.2 among medical and paramedical students regarding the impact of healthcare-associated infections on the clinical outcomes of patients. There was a statistically significant difference between the scores of both groups when they were asked whether hand hygiene was important for patient safety and the same difference was there among both groups that patients also realize that it is important for doctors to perform hand hygiene before any procedure. The perception among medical and paramedical students about hand hygiene practices has been shown in Table 4.

**Table 4:** Perception of hand hygiene practices among medical and paramedical students. * represents the p-value which is significant.

S. No.	Questions	Medical students (Mean±SD)	Paramedical students (Mean±SD)	P value
1.	In general, what is the impact of a healthcare-associated infection on a patient's clinical outcome?	6.6±0.7	5.2±1.2	0.045*
2.	What is the effectiveness of hand hygiene in preventing healthcare-associated infection?	6.1±0.8	5.8±1.1	0.342
3.	Among all patient safety issues, how important is hand hygiene at your institution?	5.9±1.2	4.9±1.2	0.004*
4.	In your opinion, how effective would the following actions be to improve hand hygiene permanently in your institution?	6.1±1.1	5.5±0.7	0.231
5.	What importance does the head of your department attach to the fact that you perform optimal hand hygiene?	6.4±1.0	5.9±1.1	0.564
6.	What importance do your colleagues attach to the fact that you perform optimal hand hygiene?	5.8±2.2	5.0±0.8	0.087
7.	What importance do patients attach to the fact that you perform optimal hand hygiene?	6.0±0.9	4.2±1.6	0.034*
8.	How do you consider the effort required by you to perform good hand hygiene when caring for patients?	4.9±1.1	4.1±0.7	0.065

Practices questionnaire

Practices of hand hygiene among students have been shown in Table 5. There was not much difference between practices of hand hygiene among medical and paramedical students regarding practices of hand hygiene but the overall practices for hand hygiene were not too good in both groups.

**Table 5:** Practices of hand hygiene among students.

S. No.	Questions	Medical students (Mean±SD)	Paramedical students (Mean±SD)	P value
1.	On average, in what percentage of situations requiring hand hygiene do you perform hand hygiene, either by hand-rubbing or handwashing (between 0 and 100%)?	2.2±05	2.1±0.2	0.457
2.	How often do you practice hand hygiene before interacting with patients?	1.8±0.3	1.2±0.2	0.362
3.	How often do you practice hand hygiene after interacting with patients?	2.3±0.3	1.9±0.9	0.231

## Discussion

Hand hygiene is the most important and simple infection control measure in preventing the transmission of hospital-acquired infections, as the hands of healthcare workers are the most common mode of transmission of pathogens from one patient to another in any healthcare setting. The “My Five Moments of Hand Hygiene” is an approach by WHO, that defines the clinical scenario where hand hygiene is to be performed by healthcare professionals in order to prevent transmission of microorganisms [4]. This approach recommends healthcare workers perform hand hygiene in these situations: before and after touching the patient, before and after any clean or aseptic procedure, and before touching the patient’s surroundings. Clinical situations in which these moments arise have also been defined by WHO. Every year on May 5, WHO celebrates World Hand Hygiene Day to create awareness of hand hygiene among the general public and healthcare workers. The MBBS and nursing undergraduates are considered to be the foundation of any healthcare system as they constitute the future doctors and nurses. So assessing and imparting them with knowledge of hand hygiene is of utmost importance. With the implementation of a competency-based curriculum as defined by the National Medical Commission in India, hand hygiene is one of the certifiable competencies in the subject of microbiology.

There have been several studies assessing the knowledge, attitude, and practices of hand hygiene among medical students but the studies in Uttarakhand are few. Analysis of the responses in our study showed that all the students had moderate knowledge of hand hygiene, similar to findings in other studies done worldwide [5-11]. Though knowledge was moderate in both groups, there were clinical scenarios where the students were not sure whether hand hygiene was to be performed or not. The students were not aware that microorganisms already present in patients are the major source of infection in healthcare settings. Medical students exhibited more such gaps in knowledge about hand hygiene though the overall score was more in medical students. Another finding in our study was that most of the participants didn’t know that hang hygiene is to be done before any clean or aseptic procedure also (medical - 22.4%, paramedical - 27.5%, Table 3). On the contrary, a study done in Germany reported that the knowledge of medical students regarding hand hygiene during their first year of MBBS was only 21% [12]. Another study done in Raichur, India showed very poor knowledge of hand hygiene among medical and nursing students and the knowledge score was better among nursing students as compared to medical students [13]. Dutta et al. showed that medical and nursing students demonstrated that only 36.6% of the participants had good knowledge about hand hygiene practices [14]. In our study, the knowledge and perception of hand hygiene were quite good in medical and nursing students. This was similar to a finding in a multicentric study in which the majority of medical and nursing students had good knowledge and attitudes toward hand hygiene, and the majority followed the proper hand hygiene practice procedures, but there was a lack of knowledge and practice regarding all the six steps of hand washing [15]. There are several other studies in India that were also done during the COVID-19 period to assess the knowledge of hand hygiene and practices among healthcare workers showing that though the knowledge was adequate, practices were not so good because of several limitations in the healthcare system including lack of proper trainings in the institute itself.

There is a limitation to this study. A comparison of different parameters could have been done among MBBS students studying in different years.

## Conclusions

Medical students and nursing students are considered to be future physicians and nurses. Knowledge of how to prevent healthcare-associated infections is an essential part of their training as medical graduates because the knowledge that they will be provided at that time will form the basis of their practices as future doctors. Knowledge gaps on hand hygiene practices among medical and paramedical students emphasize the need for induction training among them to inculcate good knowledge about hand hygiene as well as good hand hygiene practices among them.

## Appendices

Hand hygiene questionnaire

Demographic Data

1. Gender:

2. Age:

3. Medical/Paramedical student:

4. Did you receive formal training in hand hygiene in the last three years? Yes/ No

5. Have you ever heard about hand hygiene practices? Yes/ No

6. What was the source of information? Media/Other/Training in institute

Knowledge Questionnaire

1. Do you routinely use an alcohol-based hand rub for hand hygiene?

Yes/No

2. Which of the following is the main route of cross-transmission of potentially harmful germs between patients in a healthcare facility?

(a) Healthcare workers’ hands when not clean

(b) Air circulating in the hospital

(c) Patients’ exposure to colonized surfaces (i.e., beds, chairs, tables, floors).

(d) Sharing non-invasive objects (i.e., stethoscopes, pressure cuffs, etc.) between patients.

3. What is the most frequent source of germs responsible for healthcare-associated infection?

(a) The hospital’s water system.

(b) The hospital air.

(c) Germs already present on or within the patient.

(d) The hospital environment (surfaces).

4. Which of the following hand hygiene actions prevents transmission of germs to the patient?

(a) Before touching a patient.

(b) Immediately after a risk of body fluid exposure.

(c) After exposure to the immediate surroundings of a patient.

(d) Immediately before a clean/aseptic procedure.

5. Which of the following hand hygiene actions prevents transmission of germs to the healthcare worker?

(a) After touching a patient.

(b) Immediately after a risk of body fluid exposure.

(c) Immediately before a clean/aseptic procedure.

(d) After exposure to the immediate surroundings of a patient.

6. Which of the following statements on alcohol-based handrub and handwashing with soap and water are true?

(a) Handrubbing is more rapid for hand cleansing than handwashing.

(b) Handrubbing causes skin dryness more than hand washing.

(c) Handrubbing is more effective against germs than handwashing

(d) Handwashing and handrubbing are recommended to be performed in sequence.

7. What is the minimal time needed for alcohol-based handrub to kill most germs on your hands?

8. Which type of hand hygiene method is required in the following situations?

(a) Before palpation of the abdomen.

(b) Before giving an injection.

(c) After emptying a bedpan.

(d) After removing examination gloves.

(e) After making a patient's bed.

(d) After visible exposure to blood.

9. Which of the following should be avoided, as associated with increased likelihood of colonization of hands with harmful germs?

(a) Wearing jewelry.

(b) Damaged skin.

(c) Artificial fingernails.

(d) Regular use of a hand cream.

Perception

1. In general, what is the impact of a healthcare-associated infection on a patient's clinical outcome?

Very low, Low, High, Very high

2. What is the effectiveness of hand hygiene in preventing healthcare-associated infection?

Very low, Low, High, Very high

3. Among all patient safety issues, how important is hand hygiene at your institution?

Low priority, Moderate priority, High priority, Very high priority

4. In your opinion, how effective would the following actions be to improve hand hygiene permanently in your institution?

Mark only one option per row. Not effective/Very effective

(a) Leaders and senior managers at your institution support and openly promote hand hygiene.

(b) The healthcare facility makes alcohol-based handrub always available at each point of care.

(c) Hand hygiene posters are displayed at point of care as reminders.

(d) Each healthcare worker receives education on hand hygiene.

(e) Clear and simple instructions for hand hygiene are made visible for every healthcare worker

(f) Healthcare workers regularly receive feedback on their hand hygiene performance.

(g) You always perform hand hygiene as recommended (being a good example for your colleagues.

(h) Patients are invited to remind healthcare workers to perform hand hygiene

5. What importance does the head of your department attach to the fact that you perform optimal hand hygiene?

No importance

Very high importance

6. What importance do your colleagues attach to the fact that you perform optimal hand hygiene?

No importance

Very high importance

7. What importance do patients attach to the fact that you perform optimal hand hygiene?

No importance

Very high importance

8. How do you consider the effort required by you to perform good hand hygiene when caring for patients?

No effort

A big effort

Practice

1. On average, in what percentage of situations requiring hand hygiene do you perform hand hygiene, either by hand-rubbing or handwashing (between 0 and 100%)?

2. How often do you practice hand hygiene before interacting with patients?

Never

Always

Occasionally

No response

3. How often do you practice hand hygiene after interacting with patients?

Never

Always

Occasionally

No response

This content is neither created nor endorsed by Google Forms.
